# Spectral domain optical coherence tomography and B-scan ultrasonography in the evaluation of retinal tears in acute, incomplete posterior vitreous detachment

**DOI:** 10.1186/s12886-016-0242-0

**Published:** 2016-05-23

**Authors:** Solmaz Abdolrahimzadeh, Domenica Carmen Piraino, Vittorio Scavella, Barmak Abdolrahimzadeh, Filippo Cruciani, Magda Gharbiya, Santi Maria Recupero

**Affiliations:** Ophthalmology Unit, DAI Head/Neck, Azienda Policlinico Umberto I, University of Rome “Sapienza”, viale del Policlinico 155, Rome, 00161 Italy; Ophthalmology Unit, Department of Sense Organs, University of Rome “Sapienza”, viale del Policlinico 155, Rome, 00161 Italy; Polimed Beltramelli Medical Centre, via I.C. Falbo 9, Roma, 00154 Italy; Opthalmology Unit, NESMOS Department, University of Rome “Sapienza”, St. Andrea Hospital, via di Grottarossa 1035-1039, Rome, 00189 Italy

**Keywords:** B-scan ultrasonography, Complete posterior vitreous detachment, Peripheral vitreous detachment, Retinal tears, Spectral domain optical coherence tomography

## Abstract

**Background:**

The purpose of this study was to evaluate the extension and traction effects of posterior vitreous detachment (PVD) complicated with retinal tears using spectral domain optical coherence tomography (OCT) and B-scan ultrasonography.

**Methods:**

Complete ophthalmological examination, B-scan ultrasonography and spectral domain OCT were performed in patients with acute PVD and retinal tears. Vitreous detachment was classified as complete or incomplete, based on extent of posterior pole or peripheral vitreous detachment. Retinal tear location and persistent traction on the retinal flap was evaluated with B-scan ultrasonography and OCT. Categorical data were evaluated with Fisher’s exact test. Statistical significance was considered as *P* < 0.05.

**Results:**

Twenty-six eyes of 25 patients were assessed. Four eyes (15 %) presented complete PVD with detachment at the posterior pole and periphery. 22 eyes (85 %) presented incomplete PVD with detachment in the periphery. Twenty eyes presented retinal tears in the superior quadrants with respect to only 6 in the inferior quadrants (*p* = 0.006). There was a higher incidence of retinal tears in the pre with respect to post-equatorial areas (19 vs 7 eyes, *p* = 0.019). B-scan ultrasonography and OCT revealed persistent traction on the retinal tear flap in 19 and 15 eyes, respectively.

**Conclusions:**

In acute PVD, retinal tears are prevalently associated with peripheral vitreous detachment. The impact of complete or incomplete PVD can be of clinical value when evaluating patients with retinal tears.

## Background

Posterior vitreous detachment (PVD) is the separation of the posterior hyaloid membrane from the surface of the retina. According to the literature, age-related PVD begins in the form of a limited separation of the vitreous from the perifoveal retina and gradually advances to culminate in detachment of the vitreous from the optic disc in a variable period of time, which can be months or years [1]. However, partial PVD is more common than once speculated and acute symptomatic PVD does not always suggest complete detachment of the vitreous [2–5]. Binocular indirect ophthalmoscopy and 3-mirror lens biomicroscopy are the cardinal diagnostic procedures in the evaluation of acute PVD. However, the presence of a Weiss ring does not necessarily indicate complete PVD and the posterior hyaloid face may still be attached [6]. Furthermore, the Weiss ring can have morphological diversity as it can be divided or lost during the process of vitreous separation. The clinical detection of the Weiss ring can indicate that the posterior vitreous is detached from the optic disc but cannot give information on the status of the peripheral vitreous. Optical coherence tomography (OCT) has provided new perspectives with high–resolution imaging of the retina and vitreoretinal interface and can identify fine linear signals, indicating vitreous attachment, on the peripapillary, foveal, perifoveal and midperipheral areas [1, 7–9]. B-scan ultrasonography is fundamental in establishing PVD in the peripheral retina or in cases with opaque optical media like haemorrhage and cataract [10–12].Therefore, the extent of PVD can be better established with a combination of both optical coherence tomography (OCT) and B-scan ultrasonography [1, 7, 8, 13].

Retinal tears are among the complications of irregular detachment of the posterior vitreous from the retinal surface [4]. Some authors have reported that they occur as a complication of age-related PVD following acute symptomatic vitreopapillary separation [14–16]. Retinal tears are frequently located on the anterior limit of the PVD, also called the vitreous base, which can range from the equator to the ora serrata. They have been reported in 8–22 % of patients [17–20].

Some studies in the literature have evaluated the risk of delayed retinal breaks following PVD, in order to establish guidelines for the follow up of patients. However, there are no studies where the extension and traction effects of vitreous detachment as shown by B-scan ultrabiomicroscopy and OCT are evaluated. The scope of this study was to investigate if vitreous detachment is complete or incomplete in the acute phase of PVD complicated with retinal tear formation and whether the detachment involves the posterior or peripheral vitreous.

## Methods

Patients with acute, symptomatic PVD and retinal tears were examined at the retina center of the Ophthalmology Unit of the University of Rome, “Sapienza”. All patients gave informed consent to inclusion in the research, which was given institutional review board approval, and the study was conducted in accordance with Tenets of the Declaration of Helsinki.

A careful history of the patients was obtained to establish whether the symptoms (visual floaters and/or photopsia) were of recent onset. Exclusion criteria were: symptoms of more than 1 week duration, history of recent eye trauma, eye surgery, eye disease, high myopia, media opacity due to corneal or lens opacity and narrow pupil dilatation. The comprehensive ophthalmological examination undertaken by the retina specialist included visual acuity measurement with Snellen charts, slit lamp examination, vitreous biomicroscopy, indirect ophthalmoscopy and 3-mirror lens biomicroscopy of the retina. All patients underwent B-scan ultrasonography and spectral domain OCT in order to establish the location and extension of vitreous detachment. PVD was classified as complete or incomplete and as involving the posterior pole or periphery. Furthermore, traction effects of PVD on retinal tear flaps were evaluated with both instruments.

B-scan ultrasonography was performed by one experienced investigator, D.C.P., using the Cinescan S (Quantel Medical, Clermont-Ferrand, France) with a 10 MHz and 20 MHz probe. The examination technique was adopted from a method previously reported by Josè Lorenzo Carrero [4, 13]. The patients were placed supine on a reclining chair. Examination was performed with the 10MHZ probe on the eyelids. Then following topical anesthesia, the 20MHZ probe was positioned on the ocular surface through 2.5 % methylcellulose. The dB gain was adjusted when using each probe to give the finest images of the ocular structures. The quality of the images obtained using each probe was assessed with particular reference to the vitreoretinal interface at the posterior pole, the peripheral vitreous and the retinal tears. Kinetic examination was performed by evaluating vitreous movement during voluntary motion of the eye while the probe was kept immobile according to the method previously described [4].

Spectral domain OCT (Spectralis Family Acquisition Module, V 5.1.3.0; Heidelberg Engineering) with Heidelberg Eye Explorer (V 1.6.2.0; Heidelberg) was used to obtain images by one experienced investigator, V.S. All patients were dilated with tropicamide 1 % (Visufarma, Italy) and phenylephrine hydrochloride 0.5 % (Visufarma, Italy). Single-line vertical and horizontal 100-frame line scans, 50-frame (20° x 15° 19- line) raster scans, and 60-frame radial scans were taken of the posterior pole to obtain an optimal view of the vitreous.

Retinal tears were studied by instructing patients to gaze in the direction of the tear. The operators were aware of the results of the fundus examination before performing the test. 100-frame line scans and, where possible according to the localization of the tear, 50-frame raster and radial scans were performed along the retinal tear and its borders.

Retinal laser treatment for retinal tears was performed in all eyes. Fundus examination to evaluate laser treatment was carried out at 1 and 3 months from treatment.

Categorical data were evaluated with Fisher’s exact test. Statistical significance was considered as *P* < 0.05.

## Results

Twenty-six eyes of 25 patients, 9 male and 16 female, aged 45 to 71 years (mean 60.5 +/− 7.58) were included in the study. The mean spherical equivalent of subjective refraction error was −0.37 diopters (range −3.25 to + 2.50). Best-corrected visual acuity of patients was 20/20 in 23 eyes and 20/40 in 3 eyes where there was vitreous cellularity.

**Fig. 1:**
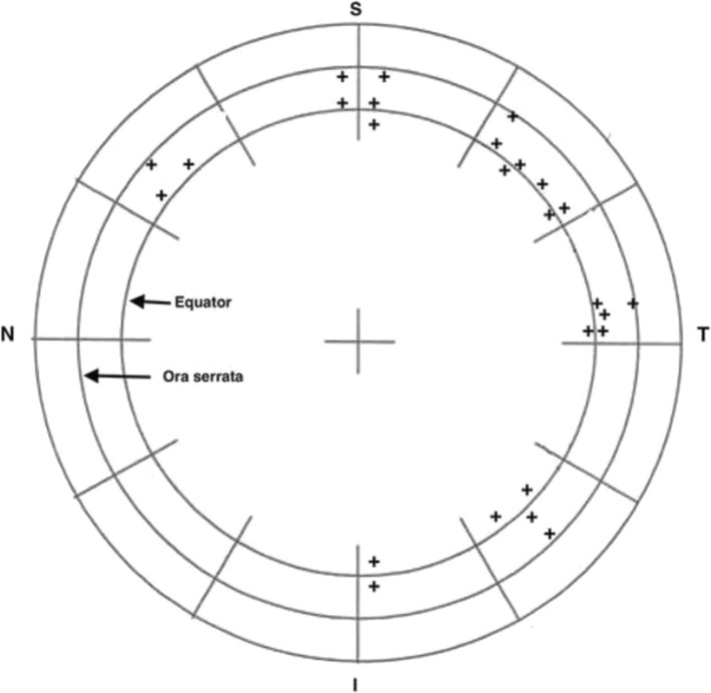
The localization of retinal tears in all eyes. Each retinal tear is represented by the sign +

**Fig. 2:**
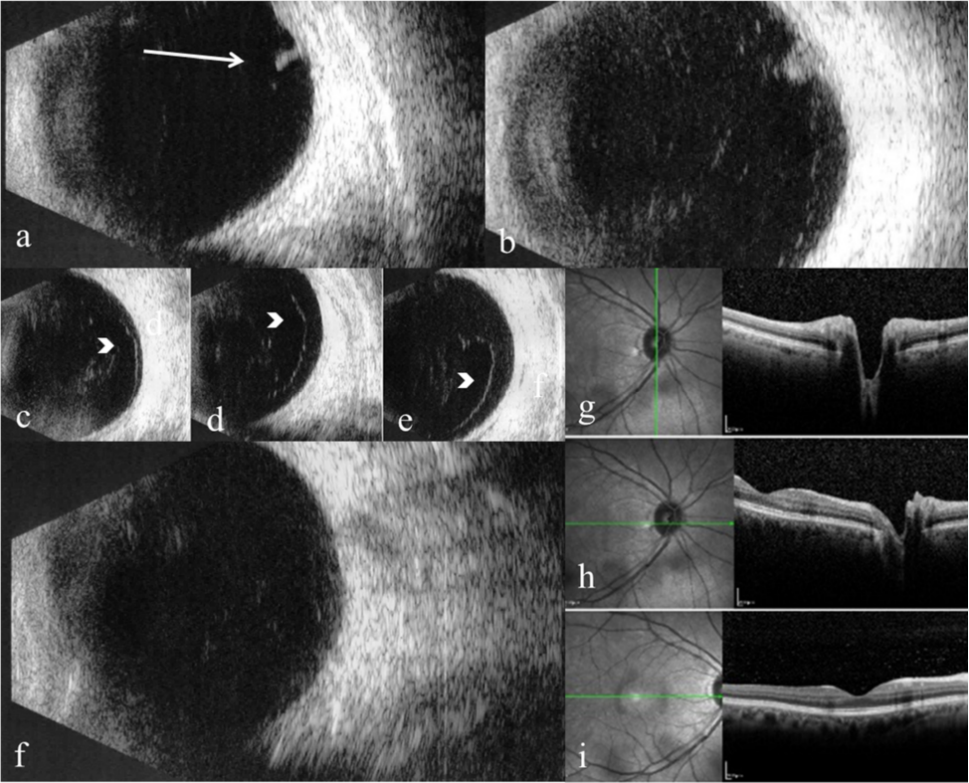
B-scan ultrasonography and optical coherence tomography showing extent of vitreous detachment. B-scan ultrasonography image showing retinal tear (arrow) (**a**); retinal tear with vitreo-retinal traction (**b**); anterior vitreous detachment (arrowhead) (**c**, **d**, **e**); posterior vitreous attachment (**f**); and optical coherence tomography images showing vitreous attached at the macula and optic nerve (**g**, **h**, **i**)

**Fig. 3:**
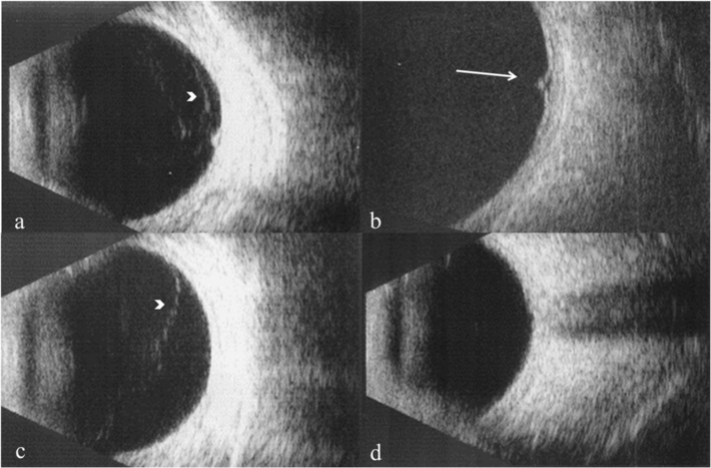
B-scan ultrasonography showing extent of vitreous detachment. Retinal tear with vitreo-retinal traction (**a**); retinal tear (arrow) (**b**); anterior vitreous detachment (arrowhead) (**c**); and posterior vitreous attachment (**d**)

**Fig. 4:**
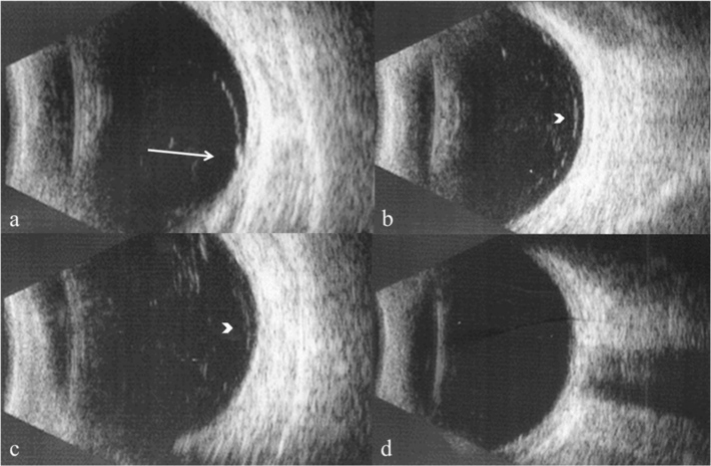
B-scan ultrasonography showing extent of vitreous detachment. Retinal tear with vitreo-retinal traction (arrow) (**a**); anterior vitreous detachment (arrowhead) (**b**, **c**); and posterior vitreous attachment (**d**)

The location of the retinal tears was post-equatorial in 7 eyes, pre-equatorial in 13 eyes and in the extreme pre-equatorial periphery in 6 eyes. The distribution of retinal tears in the retinal quadrants, in general agreement with previous reports on retinal tears, is shown in Fig. 1 [17–21]. There was a higher incidence of retinal tears in the superior quadrants and those localised in the prequatorial region (*p* = 0.006 and *p* = 0.019, respectively). There were no cases of frank vitreous hemorrhage, however, in five eyes the vitreous body presented cellularity and in these cases the retinal tears were localized in the pre-equtorial region. Complete PVD was observed with B-scan ultrasonography in only four eyes (15 %), which showed both posterior pole PVD and peripheral PVD. However, in three of these cases OCT did not show posterior pole PVD due to the technical limitation of the instrument as the posterior border of the detached hyaloid had moved very anterior to the retinal plane and could not be visualized. Ultrasonography showed that vitreous separation was only peripheral in 22 eyes (85 %) and the posterior pole was still attached. Fine details of residual perifoveal and peripapillary vitreous adherence in 2 eyes were shown with OCT, which had not been evidenced with B-scan ultrasonography (Figs. 2, 3 and 4).

**Fig. 5:**
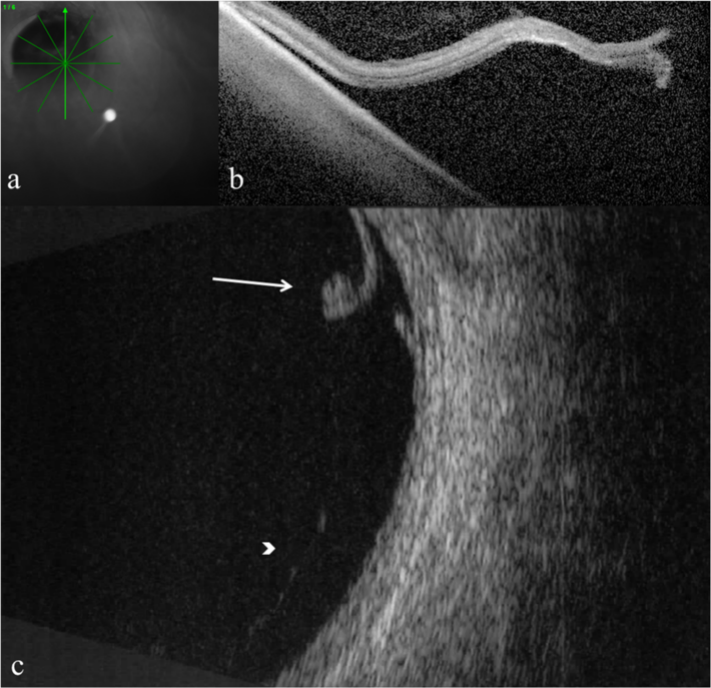
Optical coherence tomography and B-scan ultrasonography showing retinal tear and vitreo-retinal traction. Optical coherence tomography radial scan and cross-sectional images (**a**, **b**); and 10 MHz B-scan ultrasonography image (**c**). Arrow indicates retinal tear and arrowhead indicates vitreo-retinal traction

**Fig. 6:**
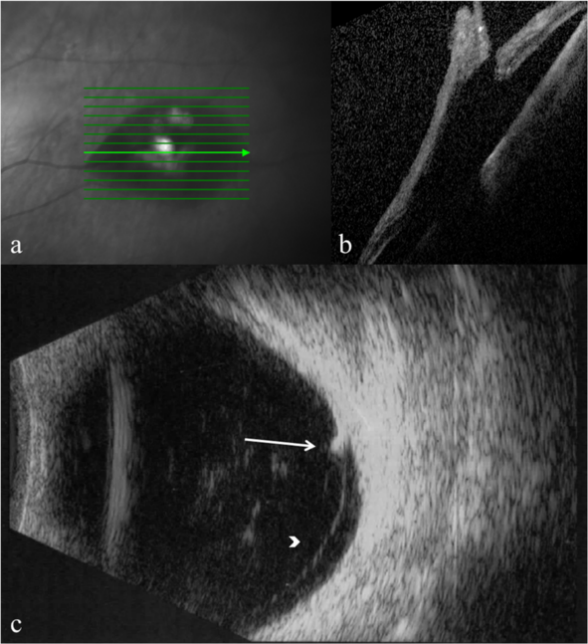
Optical coherence tomography and B-scan ultrasonography showing retinal tear and vitreo-retinal traction. Optical coherence tomography raster scan and cross-sectional images (**a**, **b**); and 10 MHz B-scan ultrasonography image (**c**). Arrow indicates retinal tear and arrowhead indicates vitreo-retinal traction

**Fig. 7:**
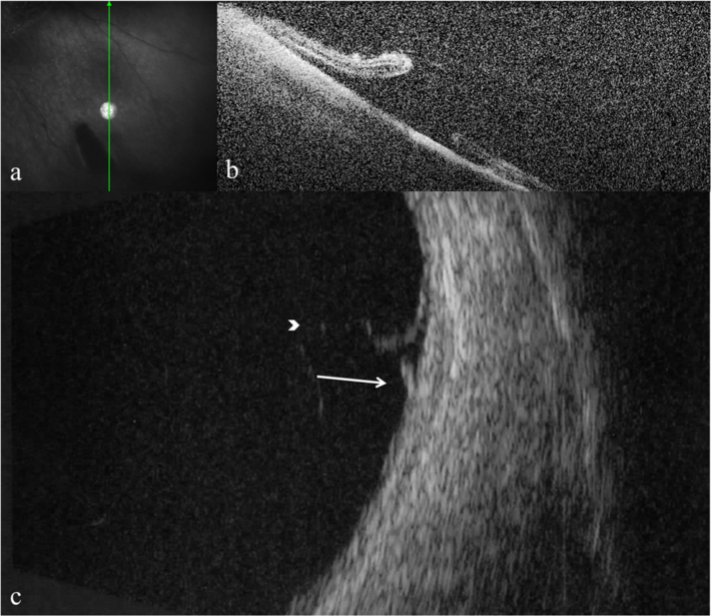
Optical coherence tomography and B-scan ultrasonography showing retinal tear and vitreo-retinal traction. Optical coherence tomography line scan and cross-sectional images (**a**, **b**); and 10 MHz B-scan ultrasonography image (**c**). Arrow indicates retinal tear and arrowhead shows vitreo-retinal traction

Persistent vitreous traction on the flap was observed in 19 eyes with ultrasonography and in 15 eyes with OCT, where scans could be obtained (Figs. 5, 6 and 7).

Fundus examination at 1, and 3 months from treatment did not show further retinal tears. In one patient with a pre-equatorial retinal tear and vitreous cellularity, retinal detachment occurred at month 1 and required surgical management.

## Discussion

The present study showed that in 85 % of cases of acute symptomatic PVD with retinal tears, vitreous detachment was incomplete and retinal tears were associated with peripheral PVD.

Various studies in the literature describe PVD based on ophthalmoscopic evidence, however, OCT allows a more precise classification of vitreous detachment of the posterior pole, which has been divided in stages based on location in the peripapillary, foveal, perifoveal, and midperipheral areas [7]. B-scan ultrasonography has been used to study the peripheral vitreous, precisely because the ophthalmoscopy finding of the Weiss ring indicates the separation of the posterior vitreous from the optic disc, but not necessarily from the peripheral retina [4]. To the best of our knowledge there are no studies performed with the concurrent use of ultrasonography and OCT to study both the posterior and peripheral vitreous in acute PVD complicated with retinal tears.

Josè Lorenzo Carrero recently reported on incomplete PVD using kinetic ultrasound examination [4]. He suggested that incomplete PVD may accommodate different forms like incomplete posterior or incomplete peripheral PVD and this may modify the common progression of vitreous detachment leading to different clinical complications [4]. Indeed, retinal tears were observed in 85 % of our cases with incomplete PVD where the detachment was chiefly peripheral and did not involve the posterior pole.

The presumed pathogenesis of retinal tears is believed to be excessive traction of the posterior vitreous cortex on areas of firm vitreoretinal adhesion. Anomalous PVD, where vitreous liquefaction takes place before adequate reduction of vitreoretinal adhesions leading to retinal tears, has been described by Sebag [22]. Byer reported that in some eyes, distinct, microscopical, pathological vitreoretinal adhesions exist which are not clinically visible and retinal tears can occur following sudden traction on these areas [23]. In the present study, persistence of fine vitreous attachment on the retinal tear flap was evidenced in 19 and 15 eyes with B-scan ultrasonography and OCT, respectively. It is thought that retinal tears result from dynamic forces created during ocular movement causing opposing tractional forces on the minute vitreoretinal adhesions. It is reasonable to hypothesize that in incomplete peripheral PVD, the vitreous could be pulled posteriorly to the plane of detachment at the peripheral site of vitreous separation, causing traction on vitreoretinal adhesions, which lead to retinal tear formation.

The limit of our study is the small number of eyes, thus, our results must be interpreted with caution. Furthermore, with the advent of swept source OCT the interface of the vitreous can be studied from the macula up to the periphery [24, 25]. The present study evaluated the vitreous with B-scan ultrasonography and OCT only in the acute stage of retinal tear formation but the extension of PVD and the presence of persistent vitreous traction on the retina over time can be clinically important and future longitudinal studies may establish if eyes with tenacious attachment at the posterior pole are a liability for new tears [26, 27]. This could give further clinical information on the impact of complete or incomplete PVD when evaluating risk in patients with retinal tears. Furthermore, studies on larger patient populations, treated with laser therapy for retinal tears, could indicate retinal detachment risk after specific treatment in relation to vitreous attachment/detachment morphology in the area of the retinal tear and indicate appropriate intervals for follow up. Moreover, with the advent of enzymatic vitreolysis for focal vitreo-macular traction, [28, 29] increased awareness of traction mechanisms might lead to new therapeutic options in specific cases. Further studies on larger patient populations are warranted.

## Conclusions

In this study we investigated the extension of acute posterior vitreous detachment in 26 eyes with retinal tears evaluated with B-scan ultrasonography and optical coherence tomography. In the acute phase of vitreous detachment, retinal tears are prevalently associated with incomplete, peripheral vitreous detachment. The impact of complete or incomplete PVD can be of clinical value when evaluating patients with retinal tears.

## Abbreviations

OCT, optical coherence tomography; PVD, posterior vitreous detachment
